# Test Format and Local Dependence of Items Revisited: A Case of Two Vocabulary Levels Tests

**DOI:** 10.3389/fpsyg.2021.805450

**Published:** 2022-01-28

**Authors:** Hung Tan Ha

**Affiliations:** School of Foreign Languages, University of Economics Ho Chi Minh City (UEH), Ho Chi Minh City, Vietnam

**Keywords:** vocabulary levels test, rasch, local independence, multiple-choice, matching

## Abstract

Local item dependence (LID) is one of the most critical assumption in the Rasch model when it comes to the validity of a test. As the field of vocabulary assessment is calling for more clarity and validity for vocabulary tests, such assumption becomes more important than ever. The article offers a Rasch-based investigation into the issue of LID with the focus on the two popular formats of Vocabulary Levels Tests (VLT): multiple-choice and matching. A Listening Vocabulary Levels Test (LVLT) and an Updated Vocabulary Levels Test (UVLT) were given to a single cohort of 311 university students in an English as a Foreign Language (EFL) context. The analyses of raw score and standardized residuals correlations were conducted. The findings found no relationship between the 4-option, multiple-choice format of the LVLT and item local dependence. However, results from score and standardized residuals correlations analyses showed a strong link between the 3-item-per-cluster, matching format and item local dependence. The study calls for greater attention to the format of future vocabulary tests and support the use of meaning-recall formats in vocabulary testing.

## Introduction

The field of vocabulary assessment is moving forward at full speed, with several issues have been raised and numerous solutions are also being suggested and considered (Schmitt et al., 2020). Especially, the years of 2020 and 2021 have witnessed the heated debate between researchers who are calling for more attention to the issues regarding the validity of vocabulary tests (Gyllstad et al., 2020; McLean, 2021; McLean and Stoeckel, 2021; Stewart et al., 2021; Stoeckel et al., 2021) and scholars who believe such so-called threats are not even worth being the topic for discussion (Laufer, 2021; Webb, 2021).

One of the mentioned problems is related to the format of vocabulary tests. Concerns have been raised regarding the strategic guessing effect of a meaning-recognition test form, the 4-option, multiple-choice format, as well as how it may inflate students’ vocabulary score and damage reliability of studies which employed such tests (Stewart et al., 2021; Stoeckel et al., 2021). However, it struck me strange that only the multiple-choice format was able to receive such privilege, and that the matching format of vocabulary tests, which is of equal age and popularity, was nearly left forgotten.

Issues with the matching format often lies with the local item dependence (LID). Although several studies have confirmed the relationship between LID and the matching format of vocabulary tests (Kamimoto, 2014; Culligan, 2015; Daly, 2019), they only focused on Schmitt et al. (2001) Vocabulary Levels Test (VLT), which could be said to be rather out-of-date, and left alone the Updated Vocabulary Levels Test (UVLT; Webb et al., 2017), which have been widely used by teachers and scholars in recent years. Moreover, questions regarding whether LID affect the quality of the other test format of vocabulary tests, the multiple-choice, still remained unanswered.

Therefore, the present study was conducted to re-examine the relationship between LID and the popular test formats in vocabulary assessment: multiple-choice and matching, with the emphasis on the two newest VLT: The Listening Vocabulary Levels Test (LVLT; McLean et al., 2015; Ha, 2021) and the UVLT (Webb et al., 2017).

## Literature Review

### Local Item Dependency in Testing

LID is one of the most important assumptions in the Rasch model and all Item Response Theory (IRT) models, which is often seen as an evidence of a test’s validity. The idea behind local dependence of test items is that different items in a test should not be closely related to each other. When a correct or incorrect answer to a certain item led to a correct or incorrect response to another item, any statistical analysis based on the students’ performance on that test would be unreliable or even misleading (Baghaei, 2007; Aryadoust et al., 2021). This is simply because if several items are locally dependent, they form a so called “polytomous super-items” (Baghaei, 2007, p. 1105), the set of items that responses to a common stimulus. These sets of super-items act as dimensions themselves which may cause the inflation of reliability and false impression of the quality of the test (Baghaei, 2007). The investigation of item local dependence could be seen to be as important as the examination of multicollinearity in linear regression analysis (Aryadoust et al., 2021).

Since local dependence makes test data too predictable, some researchers often relates item local dependence with overfit statistics, and some even use the detection of overfit as a way of investigating item local dependence (Webb et al., 2017). However, not every scholar holds the same perspective (Aryadoust et al., 2021). The most widely accepted method of examining local dependence is the investigation of the correlations between standardized residuals or raw score residuals (also known as Q3 coefficient) (Yen, 1984, 1993; Wright, 1996; Chen and Thissen, 1997; Lee, 2004; Liu and Maydeu-Olivares, 2013; Christensen et al., 2017; Fan and Bond, 2019; Aryadoust et al., 2021; Linacre, 2021).

### Local Item Dependence and Vocabulary Tests

Local dependence of test items recently attracted the attention of vocabulary linguists, especially when it came to the development and validation of VLT (McLean and Kramer, 2015; Webb et al., 2017; Ha, 2021). Popular VLT in the field mainly employ two formats: the 4-option, multiple-choice format (McLean et al., 2015; McLean and Kramer, 2015; Ha, 2021) and the 3-item-per-cluster, matching format (Schmitt et al., 2001; Webb et al., 2017). Examples of such test items are illustrated below:

An example of a 3-item cluster in the VLT (Schmitt et al., 2001).

**Table:** 

*1 business*	
*2 clock*	*_____ part of a house*
*3 horse*	*_____ animal with four legs*
*4 pencil*	*_____ something used for writing*
*5 shoe*	
*6 wall*	

An example of a 3-item cluster in the UVLT (Webb et al., 2017).

**Table:** 

	*bar*	*conversation*	*neighbor*	*rain*	*rubbish*	*shirt*
*person who lives nearby things that are thrown away*						
*type of clothing*						

An example of a test item in the NVLT (McLean and Kramer, 2015).

2. *stone: She sat on a* ***stone*** (visible on the answer sheet).
a. *hard thing*
b. *kind of chair*
c. *soft thing of the floor*
d. *part of a tree*

An example of a test item in the Vietnamese LVLT (McLean et al., 2015; Ha, 2021).

2. *[stone, she sat on a stone]* (This is what the learners hear and, therefore, is invisible on the answer sheet).

(The options in LVLTs are typically presented in the test takers’ first language, in this case for example, Vietnamese).

a. *viên đá/tång đá*
b. *cái ghế*
c. *tấm thåm*
d. *cành cây.*

When Schmitt et al. (2001 p. 61). published the VLT in 2001, they approached the issue of test items’ local dependence with care and argued that “independence stems not only from the item format itself, but also from the examinees’ test-taking behavior”. They pressed that there was a certain degree of item independence within the clusters and that the test format itself should not be the cause for concern (Schmitt et al., 2001). Schmitt et al. (2001) carried out a Rasch analysis on the test takers’ performance and investigated the correlations between standardized residuals. While their analysis found “no evidence of dependence among the items in a cluster” Schmitt et al. (2001, p. 61), called for further investigation into the issue. 16 years later, Webb et al. (2017) introduced a new version of the VLT, the UVLT, with major improvements with regard to the test’s content, however, the 3-item-per-cluster matching nature of the test remained unchanged. In their validation study, Webb et al. (2017, p. 46) examined item overfit and standardized residual correlations of the test items to investigate test’s LID. Although considerable correlations were found (0.69, 0.67, and 0.61), “none of them were presented in the same cluster”, leading to the claim that “the new forms of the VLT items may be acceptable in terms of local independence” (Webb et al., 2017, p. 46).

On the contrary, McLean and Kramer (2015) took extreme caution with item local dependency in their attempt to improve the VLT. They criticized the old matching format, saying that it was the main cause of local independence and then expressed clear preference toward another one that would be free from the issue, the multiple-choice format. However, no empirical evidence concerning whether the multiple-choice test format would lead to LID was given.

In fact, the relationship between local dependency and the matching format of the VLTs have been discussed at length in Kamimoto (2014), Culligan (2015), McLean and Kramer (2015), and Daly (2019). However, these mentioned studies only looked at the Schmitt et al. (2001) VLT. After Webb et al. (2017) published their UVLT accompanied with a Rasch-based validation study in 2017, the discussion on the problem seemed to cool down and not many researches revisited the topic, leaving questions regarding the LID of the test untouched. According to Fulcher and Davidson (2007), cited in Schmitt et al. (2020, p. 113), “Validation is seen as an ongoing process, and so tests can never be ‘validated’ in a complete and final manner.” Therefore, it is not only necessary but also crucial to keep revisiting the validity of existing tests, especially for popular ones.

### The Present Study

The common justification that both Schmitt et al. (2001) and Webb et al. (2017) used to argue their tests out of LID, even when considerable correlations were detected between item standardized residuals, was that those item pairs were not found in the same clusters.

The present study was conducted to revisit the issue from a Rasch-based perspective by using the same research methodology applied in the validation studies of both the VLT (Schmitt et al., 2001) and UVLT (Webb et al., 2017). The ultimate goal of the research was to confirm the claim made Schmitt et al. (2001) and Webb et al. (2017) regarding the residual correlations between items from the same clusters. Another aim of the study was to compare the LID between the two popular formats of vocabulary test among a single, large cohort of participants, which have never been done before.

In particular, the research seeks to answer the following research questions:

1. *Is there any significant residual correlations between test items of the two formats?*
2. *If there are significant residual correlations between test items, are they between tests items of the same clusters?*
3. *Which test format, multiple-choice or matching, would be more likely to result in item local dependence for a VLT?*

## Methodology

### Participants

The present study reported data from 311 Vietnamese second-year, non-English majors (96 males and 215 females) enrolled in a Level 4 Business English course at a highly ranked public university in southern Vietnam. Convenience sampling was applied and participants were selected based on their availability and willingness (Creswell, 2012). The participants were students in eight Level 4 Business English classes for which the researcher was responsible. The total number of students in these classes were 320 (40 students per class), 9 out of which did not took part in the study. All the participants were native Vietnamese and none of the them had spent more than one year in a country where English is the official language. All the participants had gone through 9 years of English education at elementary, middle and high schools and passed the first three levels of compulsory business English course at their university, suggesting an average English proficiency level of B1.

### Procedures

This study involved the administration of the two VLT: the UVLT (form B) (Webb et al., 2017) and Vietnamese LVLT (Ha, 2021). The two VLT were reported to have satisfied major validation requirements in Wright and Stone (1999) comprehensive framework which consisted of reliability, separation, fit statistics, unidimensionality and local dependence.

Both tests were given to the students in the same meeting, with the LVLT being given first and then the UVLT. The two vocabulary tests were administered in pencil-and-paper format and students were given as much time as they needed to complete the tests. The LVLT was administer through classroom speakers. All the test items in the LVLT were clearly heard and no technical issues arose during the administration of those tests. All the participants took part in the research voluntarily and were well informed of the significance and purposes of the study as well as the confidentiality, anonymity and security of collected data. All the participants provided their written consent to participate in the study.

After being collected, all the 311 answer sheets were manually graded, scored dichotomously and then imported into an Excel spreadsheet where the data was checked against different data collection criteria. All the collected data satisfied the requirements for data collection and analysis. The data was then imported into WINSTEPS 5.1.5 (Linacre, 2021) for analysis.

### Analysis

Collected data were computed using WINSTEPS 5.1.5 (Linacre, 2021) based on the Rasch dichotomous model (Rasch, 1960). The study re-used some of the data reported in Ha (2021), of course, with the official agreement of the author.

## Results

### Descriptive Statistics

Tables 1, 2 present the descriptive statistics of the LVLT and UVLT. In general, both the VLT recorded similar mean score of around 60% of the maximum possible score and large standard deviations. Both tests showed very strong values of item and person separation as well as reliability, confirming their reproducibility and the ability to distinguish learners of different levels.

**TABLE 1:** Descriptive statistics of the Vietnamese LVLT.

Person	311 Input		311 Measured		Infit		Outfit	
	Total	Count	Measure	Release	IMNSQ	ZSTD	OMNSQ	ZSTD
Mean	93.1	150.0	0.88	0.22	1.00	−0.1	1.02	0.0
P. SD	23.0	0.1	1.07	0.03	0.18	1.7	0.55	1.5
Real RMSE	0.23	True SD	1.04	Separation	4.61	Person Reliability		0.96

**Item**	**150 Input**		**150 Measured**		**Infit**		**Outfit**	
	**Total**	**Count**	**Measure**	**Release**	**IMNSQ**	**ZSTD**	**OMNSQ**	**ZSTD**

Mean	192.9	311.0	−0.04	0.19	1.00	0.0	1.02	0.0
P. SD	77.7	0.2	1.80	0.17	0.11	1.8	0.31	1.8
Real RMSE	0.25	True SD	1.78	Separation	7.01	Item Reliability		0.98

*The table includes data from “A Rasch-based validation of the Vietnamese version of the Listening Vocabulary Levels Test,” by Ha (2021), Language Testing in Asia.*

**TABLE 2:** Descriptive statistics of the UVLT.

Person	311 Input		311 Measured		Infit		Outfit	
	Total	Count	Measure	Release	IMNSQ	ZSTD	OMNSQ	ZSTD
Mean	90.8	150.0	1.01	0.25	0.99	–0.2	1.16	0.1
P. SD	26.9	0.2	1.52	0.05	0.23	1.8	1.25	1.7
Real RMSE	0.26	True SD	1.50	Separation	5.84	Person Reliability		0.97

**Item**	**150 Input**		**150 Measured**		**Infit**		**Outfit**	
	**Total**	**Count**	**Measure**	**Release**	**IMNSQ**	**ZSTD**	**OMNSQ**	**ZSTD**

Mean	188.4	310.9	0.00	0.21	0.99	–0.1	1.23	0.4
P. SD	86.2	0.3	2.30	0.12	0.11	1.3	1.07	1.8
Real RMSE	0.24	True SD	2.28	Separation	9.50	Item Reliability		0.99

### Local Dependence

In order to give a clear view of test item local dependency, the correlations of both standardized residuals and score residuals, also known as Yen (1984, 1993) Q3 coefficient, of the test items of the two VLT were computed. The results of the analyses were presented in Tables 3, 4. The test items for the two tests were labeled in accordance with their level and the item number on the test form. For example, item 1000-3 belonged to the first 1,000-word level and was the test item number 3. Items from the Academic Word List were named as AWL, which only applies for the LVLT.

**TABLE 3:** Largest standardized residual correlations.

LVLT			UVLT		
Correlation	Item	Item	Correlation	Item	Item
0.53	1000-3	1000-4	1.00	1000-5	1000-6
0.46	1000-22	2000-40	1.00	1000-26	1000-27
0.35	1000-13	2000-32	0.68	1000-5	1000-7
0.34	2000-40	4000-6	0.68	1000-6	1000-7
0.34	1000-21	1000-23	0.57	1000-2	1000-19
0.33	1000-12	2000-40	0.53	2000-42	4000-98
0.32	2000-40	3000-63	0.50	1000-4	1000-5
0.32	3000-63	4000-86	0.50	1000-4	1000-6
0.30	1000-12	1000-22	0.47	5000-133	5000-145
0.27	1000-22	3000-63	0.46	1000-9	2000-52
0.27	5000-115	5000-116	0.45	1000-16	1000-18
0.27	5000-99	AWL-142	0.42	1000-11	1000-12
0.26	2000-32	3000-61	0.42	4000-98	4000-111
0.26	1000-22	4000-86	0.42	1000-28	1000-29
0.26	AWL-135	AWL-136	0.41	3000-72	4000-97
0.26	1000-8	2000-26	0.40	5000-133	5000-140
0.25	1000-7	1000-20	0.40	2000-40	2000-41
0.25	5000-113	5000-115	0.39	1000-1	1000-3
−0.27	1000-13	AWL-132	−0.50	1000-18	5000-141
−0.25	5000-117	AWL-129	−0.48	1000-3	4000-116

**TABLE 4:** Largest score residual correlations (Wendy Yen’s Q3).

LVLT			UVLT		
Correlation	Item	Item	Correlation	Item	Item
0.42	5000-115	5000-116	1.00	1000-5	1000-6
0.39	1000-3	1000-4	0.91	1000-26	1000-27
0.32	1000-2	2000-45	0.74	1000-1	1000-3
0.31	5000-113	5000-115	0.53	1000-16	1000-18
0.31	AWL-142	AWL-143	0.51	1000-2	1000-3
0.31	1000-13	2000-32	0.51	1000-6	1000-7
0.30	5000-113	5000-116	0.51	1000-5	1000-7
0.30	1000-19	1000-21	0.49	1000-14	1000-15
0.30	1000-3	2000-45	0.45	1000-28	1000-29
0.28	2000-32	3000-61	0.44	1000-12	1000-18
0.27	3000-69	5000-117	0.43	1000-4	1000-7
0.25	1000-8	1000-12	0.42	1000-4	1000-5
0.25	2000-32	4000-86	0.42	1000-4	1000-6
0.25	1000-6	1000-7	0.39	2000-40	2000-41
0.25	5000-98	5000-113	0.39	3000-73	3000-75
0.24	1000-8	2000-26	0.38	1000-2	1000-19
0.24	5000-103	AWL-126	0.37	1000-1	1000-2
0.24	2000-28	2000-35	0.37	1000-3	1000-5
0.24	1000-21	1000-23	0.37	1000-3	1000-6
−0.24	2000-36	5000-117	0.36	4000-94	4000-95

In general, there are two things that we need to set our eyes on, the degree at which the test items were correlated and positions of the correlated items. In short, if two items are correlated at around 0.70, which means that they share nearly half (0.70 × 0.70 = 0.49 = ∼50%) of their variance in common, then we should be worried about these items being locally dependent (Linacre, 2021). Moreover, if a whole chain of test items located close to each other showed considerable degrees of correlation, then that would also raise a red flag in terms of local dependence.

The analyses of both score residuals and standardized residuals correlations for the LVLT did not show any substantial violation of the mentioned aspects. In fact, results from the analyses showed that the 4-option, multiple-choice format resulted in lower correlations between residuals compared to the 3-item-per-cluster, matching format. Moreover, none of the item pairs in the LVLT were found to share more than 30% of their variance in common. And even though certain correlations were found between items that were near each other, they were more likely to represent strands rather than dimensions. All of these suggested that the 4-option, multiple-choice format would not be the cause for concern, at least in terms of item local dependence.

When the correlations between standardized residuals were computed for the students’ performance on the UVLT (Table 3), significant correlations (1.00) were found between items of the same clusters (1000-5 and 1000-6; 1000-26 and 1000-27). These findings are noteworthy since standardized residuals correlations analysis was the same technique utilized by Schmitt et al. (2001) and Webb et al. (2017) in their validation studies. According to Table 1, the whole cluster of items 1000-4, 1000-5, and 1000-6 could be said to have formed a polytomous super-item. Other clusters also showed certain degree of item dependence, such as items 1000-11 and 1000-12, 1000-16 and 1000-18, 1000-28 and 1000-29.

When Yen (1984) Q3 coefficient (Table 4) were computed for test takers’ performance on the UVLT, the problems were even more visible. Besides the sky-high correlations between the first three item pairs, it is impressive that most of the correlations found were between items of the same clusters. Some clusters were shown to contain highly correlated items, for example, 1000-1-2-3, 1000-4-5-6. All of these together signaled the presence of different underlying dimensions and a possible violation of local independence in the Rasch model.

It is worth noting that the UVLT presented higher values of item and person reliability and separation, suggesting a healthier test compared to the LVLT. However, the metrics of local dependence signaled the format of the test itself was problematic and deserved serious reconsideration.

## Discussion

The present study compared the residual correlations between test items of the two VLT to see if test format is the cause of item local dependence as well as to confirm the claims made in Schmitt et al. (2001) and Webb et al. (2017).

Results from the analyses showed that, for the UVLT that employed the matching format, both the score and standardized residual correlation analyses showed strong correlations between the same pairs of items that belonged to the same clusters. More importantly, some of these items were reported to highly correlated at greater than 0.70, signaling a potential violation of the assumption of LID.

The study was well in line with Daly (2019) which utilized a Rasch-based methodology to investigate the issue of LID in Schmitt et al. (2001) VLT. It also confirmed the findings of Kamimoto (2014) who challenged the LID of Schmitt et al. (2001) VLT through a non-rasch approach. It seemed that McLean et al. (2015) and McLean and Kramer (2015) was right when decided not to keep the matching format and use the multiple-choice format for their NVLT and LVLT. Although the multiple-choice format was also challenged for its issue concerning test takers’ strategic guessing, at least they would not have to worry about their test items being locally dependent.

The present study offers an empirical evidence for English teachers and researchers who are interested in vocabulary assessment and are considering employing vocabulary tests in their research and material or syllabus planning. If we take the current study’s findings into account, then it is true that both the multiple-choice and matching formats of vocabulary tests are receiving serious criticism concerning their validity (Gyllstad et al., 2020; Stewart et al., 2021; Stoeckel et al., 2021), leaving scholars and teachers confused of what to use for their job and their research. In fact, vocabulary tests that utilized meaning-recognition formats are being severely challenged for their reliability and validity from various aspects, and some researchers are encouraging the use of meaning-recall vocabulary tests which are believed to ensure greater reliability and validity (Stewart et al., 2021; Stoeckel et al., 2021). To be fair, I also hold that such issue of LID would never be the cause for concern if these VLT were to be presented in a meaning-recall format, the format in which students would write the definition or a direct L1 translation of a word rather than choosing the correct option.

## Conclusion

The study contributes to the serial attempts to move “the field of vocabulary assessment forward” (Schmitt et al., 2020, p. 109). Several vocabulary linguists are trying their best to raises problems that could threaten the validity of vocabulary tests and falsify research data (Gyllstad et al., 2020; Schmitt et al., 2020; McLean, 2021; McLean and Stoeckel, 2021; Stewart et al., 2021; Stoeckel et al., 2021). Those issues include the strategic guessing effect in the 4-option, multiple-choice format, the word counting unit and score interpretation methods, to name a few. The present study would suggest that if the 4-option, multiple-choice format was deemed problematic, then the matching format would not be a good replacement either.

Despite being informative, the research itself bears certain limitations. Firstly, it only dealt with one form of the UVLT (form B) and only investigated the issue of LID from one methodological perspective. Future researches should re-examine LID from a wider range of vocabulary tests and should employ different approaches to provide a holistic view of the issue. Secondly, due to space limitation, the research could have flagged possible violations of LID assumption of the UVLT, but was not able to go deeper and explore how these violations could possibly have led to problems in the estimation of learners’ vocabulary knowledge or a linked inflation of learners’ language ability like reading and listening comprehension. Therefore, further investigations on these aspects are especially welcomed.

## Data Availability Statement

The raw data supporting the conclusions of this article will be made available by the authors, without undue reservation.

## Ethics Statement

The studies involving human participants were reviewed and approved by University of Economics Ho Chi Minh City. The participants provided their written informed consent to participate in this study.

## Author Contributions

The author confirms sole responsibility for the following: study conception and design, data collection, analysis and interpretation of results, and manuscript preparation.

## Conflict of Interest

The author declares that the research was conducted in the absence of any commercial or financial relationships that could be construed as a potential conflict of interest.

## Publisher’s Note

All claims expressed in this article are solely those of the authors and do not necessarily represent those of their affiliated organizations, or those of the publisher, the editors and the reviewers. Any product that may be evaluated in this article, or claim that may be made by its manufacturer, is not guaranteed or endorsed by the publisher.
